# Endoscopic Ultrasound for Nodal Staging in Patients with Resectable Cholangiocarcinoma

**DOI:** 10.3390/jcm14217545

**Published:** 2025-10-24

**Authors:** David M. de Jong, Lydi M. J. W. van Driel, Sundeep Lakhtakia, Mohan Ramchandani, Sana Fathima Memon, Abhishek Tyagi, Parathasarathy Kumaraswamy, Shreeyash Modak, Anuradha Sekaran, Marco J. Bruno, Duvvur Nageshwar Reddy, Hardik Rughwani

**Affiliations:** 1 Department of Gastroenterology and Hepatology, Erasmus MC University Medical Center, 3015 GD Rotterdam, The Netherlands; 2 Department of Gastroenterology, Asian Institute of Gastroenterology (AIG Hospitals), Hyderabad 500032, India; 3 Department of Artificial Intelligence, Asian Institute of Gastroenterology (AIG Hospitals), Hyderabad 500032, India; 4 Department of Surgery, Asian Institute of Gastroenterology (AIG Hospitals), Hyderabad 500032, India; 5 Department of Pathology, Asian Institute of Gastroenterology (AIG Hospitals), Hyderabad 500032, India

**Keywords:** cholangiocarcinoma, endoscopic ultrasound, lymph node

## Abstract

**Background**: Lymph node (LN) involvement is a negative prognostic factor for patients with cholangiocarcinoma (CCA). Preoperative assessment of the LN could potentially aid therapy decision making. Endoscopic ultrasound (EUS) can be used to sample suspicious LN. The aim of this study was to evaluate the clinical impact of EUS for suspicious LN in patients with presumed resectable CCA. **Methods**: In this single-center cohort study, patients with potentially resectable CCA who underwent preoperative linear EUS between 2019 and 2024 were retrospectively included. The primary aims were the percentage of malignant LN detected and the clinical impact of EUS, which was defined as the percentage of patients who were precluded from surgical exploration due to pathologically confirmed LN metastases found with EUS tissue acquisition (EUS-TA). The secondary aim was the complication rate of EUS-TA. **Results**: A total of 135 patients were included, of whom 12 (8.9%) had intrahepatic CCA (iCCA), 65 (48.1%) had perihilar CCA (pCCA), 13 had (9.6%) middle bile duct CCA (mCCA), and 45 (33.3%) had distal CCA (dCCA). Across 148 EUS procedures, 139 LNs were identified, and EUS-TA was performed on 63 LNs among 55 patients. LN metastases were detected by EUS-TA for iCCA, pCCA, mCCA, and dCCA, in 25%, 6.2%, 15.4%, and 4.4%, respectively. EUS and EUS-TA influenced surgical work-up for iCCA, pCCA, mCCA, and dCCA in 25%, 1.5%, 15.4%, and 0.0%, respectively. No complications associated with EUS were noted. **Conclusions**: Preoperative EUS for nodal staging had an important clinical impact in patients with presumed resectable iCCA and mCCA, but less for pCCA and dCCA. Further prospective studies should investigate whether systematic nodal staging with EUS could improve preoperative decision making even further.

## 1. Introduction

Cholangiocarcinoma (CCA) is an uncommon malignancy originating from the bile ducts, classified into intrahepatic CCA (iCCA), perihilar CCA (pCCA), and distal CCA (dCCA) subtypes [1]. For all subtypes, resection is the only curative treatment option, yielding a 5-year survival rate of 30.4% in iCCA, 17% in pCCA, and 11% in dCCA [2,3,4]. From a surgical point of view, CCA of the middle bile duct (mCCA) has gained acceptance as a separate anatomic subtype of dCCA due to the implications for surgical management, including the need for only bile duct resection rather than pancreato-duodenectomy, which is typically required for dCCA [5,6]. For all subtypes, various histopathological factors, including tumor stage, resection margins, and differentiation grade, are linked to decreased postoperative survival.

Lymph node (LN) metastasis is a key prognostic factor in CCA [2,3,4]. According to the 8th edition of the American Joint Committee on Cancer (AJCC) staging manual, nodal (N) status is based on the number of positive regional LN, while positive extraregional LN are classified as distant metastases (Table 1) [7]. Regional LN involvement significantly reduces survival after resection: for iCCA, median overall survival drops from 45 to 18 months [2]; in pCCA, median overall survival drops from 34 to 15 months [8]; in mCCA, median overall survival drops from 52 to 25 months [9]; and in dCCA, median overall survival drops from 55.5 to 27.5 months [10]. However, conventional cross-sectional imaging such as computed tomography (CT) and magnetic resonance imaging (MRI) show limited accuracy in differentiating malignant from non-malignant LN and ranges from 66 to 76.5%, underscoring the need for improved LN staging techniques [11,12,13,14].

Recent studies on preoperative endoscopic ultrasound (EUS) with tissue acquisition (EUS-TA) have exhibited promise in LN assessment for all CCA subtypes [15,16,17,18,19,20,21,22,23]. Adequate preoperative LN staging through EUS-TA in resectable cholangiocarcinoma patients has shown potential in sparing patients from an unnecessary laparotomy [15,16,17,18,19,20]. While regional LN metastases are only a relative contraindication for resection, patients with extraregional LN metastases are not eligible for resection. Although the latest European Society of Gastrointestinal Endoscopy (ESGE) guidelines recommend the combination of EUS and endoscopic retrograde cholangio-pancreatography (ERCP) for biliary strictures [24], no formal statement on preoperative LN assessment by EUS has been issued. The European Association for the Study of the Liver (EASL) Clinical Practice Guidelines 2025 recommend ruling out metastatic LN by EUS in the setting of possible surgical treatment for all CCA subtypes [25,26]. Preoperative LN assessment in CCA could guide treatment decisions, including preventing surgery in case of metastatic lymph nodes or the potential use of neo-adjuvant treatments for patients with positive regional LN.

The use of preoperative LN staging by EUS in CCA has shown promise in previous retrospective studies. However, most data originate from Western populations, and the extent to which EUS-TA influences clinical decision making in other settings remains unexplored. This study addresses that gap by analyzing the diagnostic yield and therapeutic impact of EUS-TA in a large cohort of presumed resectable CCA patients from India.

## 2. Materials and Methods

### 2.1. Study Population

This single-center retrospective cohort study was conducted at the Asian Institute of Gastroenterology (AIG Hospitals), Hyderabad, India after approval by the local ethics committee (AIG/IEC—Post BH&R 60/06.2024-02). Consecutive patients with presumed resectable CCA who underwent linear EUS preoperatively were included from January 2019 to December 2024. Exclusion criteria were prior surgical treatment for CCA and unresectable CCA. Identification of patients was performed through endoscopy report databases.

### 2.2. EUS Procedure and Work-Up for Surgery

Prior to EUS, patients routinely underwent cross-sectional imaging by one or more of the following modalities: computed tomography (CT), fluorodeoxyglucose (FDG) positron emission tomography (PET) CT, fibroblast-activation-protein inhibitors (FAPIs) PET CT, or magnetic resonance imaging (MRI). Suspicious LN on cross-sectional imaging was based on a short axis diameter larger than one cm in diameter or a high standardized uptake value (SUV) max (value > 5) for PET scans.

At the discretion of the local management team, EUS was performed for one or both of the following indications: (1) assessment of the primary lesion or stricture, or (2) assessment of regional and/or extraregional LN based on cross-sectional imaging. A systematic LN assessment was not performed. The EUS procedure could be performed at the study site or one of the referring hospitals. At the study site, the procedures were performed using a linear ultrasound endoscope (GF-UCT 180, Olympus Corporation, Hachioji, Tokyo, Japan). Suspicious LNs were defined as having one or more of the following characteristics: short axis diameter > 10 mm, hypo-echoic, round shape, and clear demarcation. EUS-TA of the lesion or LN was performed at the discretion of the endosonographist. Fine-needle aspiration (FNA) or fine-needle biopsy (FNB), both 22 or 25 G-needles (Cook Medical, Bloomington, IN, USA, or Boston Scientific, Marlborough, MA, USA), was used. Rapid on-site evaluation (ROSE) was not routinely performed. LN locations were defined according to the 8th AJCC edition, if possible, based on radiology and endoscopy reports (Table 1) [7]. The decision to proceed with surgical exploration for patients with EUS-TA-confirmed positive LN was made after consultation with the hepato-biliary surgery and oncology team. Neo-adjuvant chemotherapy was not routinely considered, but on a case-to-case basis.

### 2.3. Outcome Definition

The primary outcomes were the percentage of malignant LNs found with EUS and the impact of EUS-TA on clinical decision making, analyzed overall and by subtype of CCA, which was defined as the number of patients for whom surgical exploration was withheld or postponed to facilitate neo-adjuvant chemotherapy administration, due to pathological confirmation of positive LNs with EUS-TA divided by the total number of patients who underwent EUS. The secondary outcome was EUS-TA-associated complications, according to the AGREE criteria [27].

### 2.4. Data Collection

Data collection was performed through the local electronic medical record system. Each electronic patient record was systematically reviewed. Data were collected on patient and disease demographics (age, sex, and American Society of Anesthesiologists’ Physical Status Classification System (ASA)). Data on LN identified at cross-sectional imaging (number, location, suspiciousness, and PET-avidity), EUS (number, location, suspiciousness, EUS-TA characteristics, and outcomes), and surgical procedures (number, location, and pathology outcomes) were retrospectively collected. Cross-sectional imaging was not revised.

### 2.5. Statistical Analysis

Descriptive statistics were used. Categorical and dichotomous variables were described using frequencies and proportions, while continuous data was described using medians with interquartile ranges for non-normally distributed variables and means with standard deviations for normally distributed variables. The statistical analyses were performed using R version 4.4.2.

## 3. Results

### 3.1. Baseline Characteristics

A total of 135 patients were included, with a median age of 59 years [IQR: 49.5–65.0] and the majority (69.6%) was male. Among this cohort, 12 (8.9%), 65 (48.1%), 13 (9.6%), and 45 (33.3%) patients had iCCA, pCCA, mCCA, and dCCA. Of the 135 patients, 126 (93.3%) underwent cross-sectional imaging before the EUS procedure. On imaging, regional LNs were identified in 59 patients (43.7%) and extraregional LNs were identified in 37 patients (27.4%). Information on whether LNs were suspicious or not based on cross-sectional imaging was not available. These and other baseline characteristics are described in Table 2. In the Supplementary File, separate results on FAPI PET scans are reported.

### 3.2. EUS Procedures and Impact on Clinical Decision Making

A total of 148 EUS were performed, with thirteen patients (9.6%) having more than one procedure. During 94 procedures, among 90 patients, LNs were identified (Table 3). These LNs were located at regional stations in 106 (76.3%), and at extraregional stations in 33 (23.7%) procedures. Reasons why EUS-TA was not performed are described in Table 4. EUS-TA was successfully performed on 63 LNs (45.3%) among 56 patients (41.4%), through FNA in 33 LNs, and through FNB in 30 LNs (Table 4). Of these sixty-three biopsied LNs, twelve (19.0%) were classified as malignant, fifty (79.4%) were classified as benign, and one was classified (1.6%) as an ‘atypical cell’. FNA confirmed malignancy in 7/33 (21.2%), and FNB confirmed malignancy in 5/30 (16.7%), in a total of eleven patients. For iCCA, pCCA, mCCA, and dCCA, EUS-TA of LN yielded malignancy in 25%, 6.2%, 15.4%, and 4.4%, respectively. There were no complications associated with EUS or EUS-TA.

**Figure 1:**
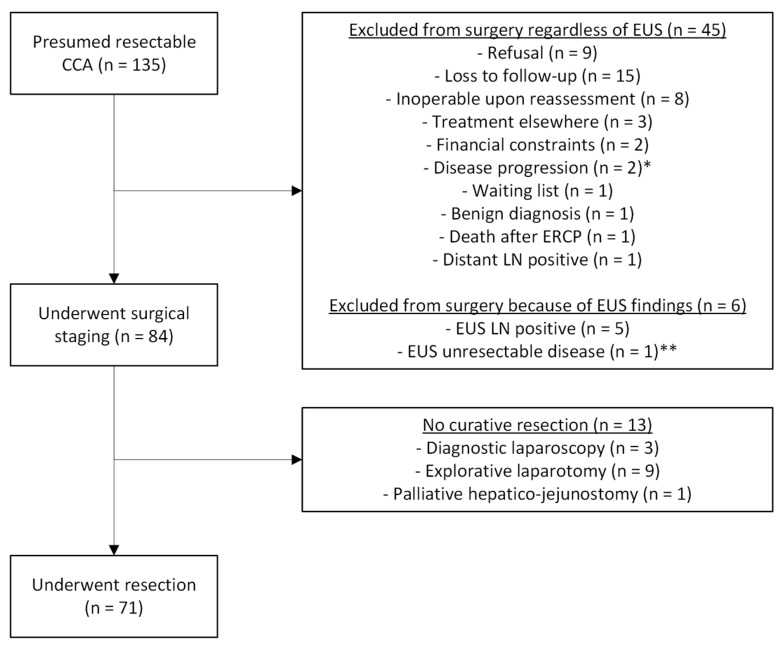
Flowchart of the included patients. * = One patient with EUS-TA proven LNM developed headache, CT showed brain metastasis; ** = Vascular encasement of the superior mesenteric artery, not seen on cross-sectional imaging.

In the 135 patients, EUS precluded surgical exploration in six patients (4.4%); in five patients (3.7%), this was because of positive LN on EUS-TA, and in one patient (0.7%), this was because of undiagnosed encasement of the superior mesenteric artery. Among the five patients with EUS-TA-confirmed LN, one received palliative chemotherapy, two patients refused, and two patients were referred to another hospital for palliative care. In addition to these six patients, in forty-five patients (33.3%), surgery was precluded due to various reasons, as shown in Figure 1. EUS influenced surgical work-up for iCCA, pCCA, mCCA, and dCCA in 25%, 1.5%, 15.4%, and 0.0%, respectively. For all subgroups, the respective flowcharts are presented in Supplementary Figures S1–S4.

### 3.3. LN Metastases Identified by EUS

EUS identified twelve LN metastases in eleven patients. Extraregional LN were found in four patients, and in one patient, regional LNs were also found. Surgery was subsequently precluded in these four patients. Regional LN metastases were found in the other seven patients, who were all worked up for resection, of which only four patients finally underwent resection. At cross-sectional imaging, no lymphadenopathy was noted in three patients. In three patients, the LN identified at EUS-TA was suspicious on cross-sectional imaging. In four patients, suspicious lymphadenopathy was described, but its specific location was insufficiently described. In another patient, only suspicious regional LNs were described, but at EUS-TA, an extraregional LN was proven malignant.

### 3.4. Surgery Procedures

After a median period of 24 days [IQR: 8.8–47], 84 patients (62.2%) underwent surgical exploration, with complete resection in 71 of the 84 patients (52.6%). In two patients (1.5%), both with pCCA, resection was precluded due to LN metastases (periportal and paraaortic LN, respectively). Among the seventy-one patients that underwent resection, LN metastases were identified in thirty-one patients (43.1%), of whom twenty-three (31.0%) had pN1 and eight (11.3%) had pN2. In eight patients (11.3%), no LNs were retrieved, defined as Nx. In six patients (4.4%), benign disease was identified without malignancy in the resection specimens. Only one patient without EUS-TA-confirmed LN metastases was administered with neo-adjuvant chemotherapy.

### 3.5. LN Metastases Missed by EUS

During surgery, no extraregional LN metastases were identified. In 30 patients, regional LN metastases that EUS did not identify were harvested. In two patients, these were identified during surgery, precluding resection; and in 28 patients, these were identified in the resection specimens. In twelve patients, no LNs were identified; in eleven patients, only regional LNs were identified; extraregional LNs were identified in three patients; and both regional and extraregional LNs were identified in one patient. In nine patients, EUS-TA was performed, yielding benign tissue, consisting of eight regional LNs and one extraregional LN. For the patients in whom EUS identified LNs, Table S1 shows the EUS findings and pathology findings of resections on the ‘missed’ LNs. Among the 64 patients with tissue-proven malignancy, after excluding those with EUS-TA-confirmed LN metastasis and including only those in whom at least one LN was harvested at surgery, the interval between the last EUS and surgery was not associated with a “missed LN metastasis” (median time: N0 group, 20 days [IQR: 10.3–35] vs. N1–2 group, 35 days [IQR: 9.5–58.8]; *p* = 0.11).

## 4. Discussion

It is crucial to identify LN metastases early on in the disease course for presumed resectable cholangiocarcinoma, as LN metastases are an established predictor for poor postoperative survival and potentially guide management decisions. Unfortunately, cross-sectional imaging has low accuracy in identifying LN. In this single-center study, we demonstrated that surgical exploration was precluded in 4.4% of patients with presumed resectable CCA, mostly by EUS-TA-showing LN metastases. Incorporating EUS for LN assessment in surgical work-up seems promising. However, a significant portion of the regional LN metastases was missed, highlighting the need for protocol optimization.

In iCCA, EUS had the highest yield precluding surgical resection in 25% after confirmation of LN metastases with EUS-TA. This is in line with the yield of two recently published studies on this subgroup, namely, 17% and 30% preclusion rate [16,17]. There are some important differences between these studies and the current one. Not only patient selection, but also the threshold of performing EUS-TA, as Malikowksi et al. performed FNA in 98.5% of identified LNs, regardless of size [16]. Unfortunately, they did not differentiate between regional and extraregional LN nor specify the location of the primary lesion. However, for iCCA, this is of great importance, as (1) differentiation between regional and extraregional LN is dependent on the location of the primary lesion, and (2) patients with extraregional LN metastases have stage IV disease precluding surgical treatment, while in regional LN metastases a case-to-case consideration is needed [7]. It is important to perform EUS-TA on all identified LNs, or at least as many as possible, as we do not have good objective criteria on which to base the decision to puncture, and interobserver agreement on any of the current criteria is low [28].

For pCCA, EUS precluded surgical work-up in only 1.5%. This is relatively low compared to the current literature [15,16,18,19]. This is probably due to the previously mentioned differences in EUS-TA threshold, since in the current study the threshold of short-axis diameter was mostly 10 mm, although exact measurements were often missing. Lowering that threshold may improve the results. Another explanation may be the differences in clinical decision making whenever regional LN metastases are present. Although it is understandable to give patients the chance to achieve resection, a recent meta-analysis gives substantiation to being more restrictive regarding direct surgical exploration and resection [29]. The 2025 EASL guidelines recommend preoperative EUS to rule out metastatic LN, while also only recommending surgical resection in N1 pCCA if it is limited to perihilar LN and the anticipated postoperative mortality is acceptable [25]. Although not routinely considered for pCCA, neo-adjuvant chemotherapy could potentially improve overall survival [30]. This might be more favorable for patients with positive regional LN. However, randomized controlled trials are needed to validate these assumptions.

In the setting of mCCA, EUS has two primary potential aims. Historically, mCCA has been incorporated in pCCA or dCCA cohorts, depending on the surgical procedure. From an endoscopic point of view, the distinction between mCCA and pCCA/dCCA is a new concept. This is the first study, albeit in a small cohort, that assessed EUS’ influence on surgical work-up for mCCA. In 15.4% of the 13 patients, surgery was precluded. In one patient, extraregional LN metastases were identified with EUS-TA, and in another patient, involvement of the superior mesenteric artery was visualized. Another complementary characteristic of EUS for mCCA is its ability to assess the proximal and distal margin. It can be difficult to assess this on cross-sectional imaging, particularly whenever a stent is already placed in the biliary tree. Whenever a stent is not in situ, EUS findings could help the surgeon when the surgical plan is discussed with the patient, as an extrahepatic bile duct resection is significantly different from a pancreato-duodenectomy regarding complication rate and overall survival post-resection.

EUS did not influence clinical decision making in any of the 45 dCCA patients. This might be because the EUS did not focus on detecting LN in these patients. Instead, EUS was performed to assess the primary lesion or acquire pathological confirmation since preoperative differentiation between dCCA and pancreatic cancer can be difficult based on cross-sectional imaging alone. This is in line with the only study reporting on preoperative EUS for dCCA, resulting in a relatively underwhelming yield in both studies [31]. However, looking at the ESGE guidelines, they state that EUS can aid in tumor staging and access to regional or distant LNs, but without any supporting literature [24]. Nonetheless, for dCCA, LN metastases are a proven predictor for decreased overall survival, underlining the potential benefit for optimizing EUS LN assessment in these patients also [32]. Therefore, the EASL 2025 guidelines recommend preoperative EUS for dCCA [25,26].

The data of this study, together with the current literature, warrants for improved preoperative assessment by EUS of the presumed resectable CCA. Although a head-to-head comparison has not been performed yet, preoperative EUS has the potential to 1) save patients from a surgical procedure that is not indicated, and 2) be more cost-effective. There are two approaches possible, of which Malikowski et al. proposed the first [16]: to perform EUS in all patients without obvious metastatic disease, which is a strategy studied in the ongoing prospective POELH-II trial in the Netherlands, including all presumed resectable iCCA, pCCA, and mCCA (Clinicaltrials.gov NCT05678218). The second option is to improve cross-sectional imaging regarding LN detection. With this second strategy, one aims to improve the yield of conventional modalities by systematic mapping, use of radiomics, or artificial intelligence. Other promising possibilities are the use of more sensitive tracers for PET CT or PET MRI scans, such as FAPI, as FDG still has an underwhelming yield in CCA [33,34].

Among the 84 patients who ultimately underwent surgical exploration, LN metastases were identified intraoperatively in 30 patients. In two cases (one regional, one extraregional), LN metastases were detected before resection, precluding surgery. In the remaining 28 patients, regional LN metastases were found in the resection specimens. It is difficult to determine whether these LNs were “missed” by EUS. Possible explanations include: (1) omission of certain LN stations due to the non-systematic nature of the EUS examination or the absence of suspicious sonographic features, (2) sampling error during EUS-TA, and (3) progression of disease during the interval between EUS and surgery. Although it is plausible that the diagnostic yield of EUS could be improved by adopting a systematic LN assessment protocol, robust evidence to support this is lacking. As shown in Table S1, sampling error during EUS-TA was likely in two cases; however, confirmation is challenging when neither endosonographers nor surgeons systematically document LN station locations. There was no statistically significant difference in the interval from EUS to surgery between patients in whom LN metastases were missed and those without LN metastases (*p* = 0.11).

Although the results of this study are promising and in line with previous publications on the topic in Western populations, some limitations need to be taken into consideration. Firstly, this is a retrospective single-center study. Data was lacking in some important variables such as several LN characteristics on cross-sectional imaging during EUS and at surgery, and the number of passes of EUS-TA; a relatively high number of patients were also lost to follow-up. Whether a specific LN was suspicious or not was often not available on cross-sectional imaging, making comparison of cross-sectional imaging with EUS difficult. It is, however, important to note that endonographers were not blinded to cross-sectional imaging results. Secondly, the indications of the EUS varies, leading to an underestimation of the potential yield of EUS when all procedures would have been focusing on LN staging. A specific reason why an LN was not targeted by EUS-TA was also missing for some patients. Third, differences in LN station nomenclature between surgeons, pathologists, and endosonographers make it difficult to determine whether LN metastases were truly missed by EUS. Also, there are different cut-offs used for ‘suspicious’ LNs for radiologists (>15 mm) and endosonographers (>10 mm), while we know that a short axis of <10 mm does not preclude LN metastasis [15]. This underlines the need for a systematic approach by radiologists, endosonographers, surgeons, and pathologists in naming LN stations identified during their respective procedures. The POELH-II trial aims to do exactly that (Clinicaltrials.gov NCT05678218).

In conclusion, this study performed in an Indian population confirms that preoperative EUS has a clinical impact in patients with presumed resectable CCA, but also highlights some factors that potentially could improve its yield by applying a systematic approach.

## Figures and Tables

**Table 1:** AJCC staging system classification regarding LN status for dCCA.

	AJCC 8th Edition		
	N1	N2	M1
iCCA left	1–3 LNM in the regional LNs (hilar, CD, CBD, HA, PV, IP or GH LNs)	≥4 LNM in the regional LNs (hilar, CD, CBD, HA, PV, IP or GH LNs)	Distant metastasis (includes LNM in the CO, PA or PC LNs)
iCCA right	1–3 LNM in the regional LNs (hilar, CD, CBD, HA, PV, PPD or PP LNs)	≥4 LNM in the regional LNs (hilar, CD, CBD, HA, PV, PPD or PP LNs)	Distant metastasis (includes LNM in the CO, PA or PC LNs)
pCCA	1–3 MLN in the regional LNs (H, CD, CBD, HA, PPD or PV)	≥4 MLN in the regional LNs (H, CD, CBD, HA, PPD or PV)	Distant metastasis (includes MLN distant to the HDL)
dCCA + mCCA	1–3 LNM in the regional LNs (PH, HA, AP, PPD, SMA)	≥4 LNM in the regional LNs (PH, HA, AP, PPD, SMA)	Distant metastasis (includes MLN in the CO, PA or PC LNs)

Nx is defined as ‘regional lymph nodes cannot be assessed’ and N0 is defined as ‘no regional lymph node metastases’. PH = porta hepatis; HA = hepatic artery; AP = anterior to the head of pancreas; SMA = superior mesenteric artery; H = hilar; CD = cystic duct; CBD = common bile duct; PV = portal vein; PP = peripancreatic; PPD = posterior pancreato-duodenal; PA = periaortic; PC = pericaval; CO = celiac; HDL = hepato-duodenal ligament; GH = gastrohepatic; IP = inferior phrenic; LNM = lymph node metastasis.

**Table 2:** Baseline characteristics of study population.

Variable	Total (*n* = 135)	iCCA (*n* = 12)	pCCA (*n* = 65)	mCCA (*n* = 13)	dCCA (*n* = 45)
Age at EUS in years, median [IQR]	59 [49.5–65.0]	52.5 [49.8–65.3]	59 [47–65]	62 [51–64]	59 [53–65]
Male sex—*n* (%)	94 (69.6)	8 (66.7)	47 (72.3)	9 (69.2)	30 (66.7)
PSC—*n* (%)	0 (0)	0 (0)	0 (0)	0 (0)	0 (0)
ASA classification—*n* (%)					
1	3 (2.2)	1 (8.3)	2 (3.1)	0 (0)	0 (0)
2	100 (74.1)	8 (66.7)	49 (75.4)	12 (92.3)	31 (68.9)
3–4	30 (22.2)	3 (25.0)	14 (21.5)	1 (7.7)	12 (26.7)
Missing	2 (1.5)	0 (0)	0 (0)	0 (0)	2 (4.4)
Total bilirubin in mg/dL, median [IQR]	6.9 [2.2–13.0] ¥	1.0 [0.7–1.95]	8.7 [3.2–14.8]	9.9 [1.8–12.8]	6.7 [2.4–12.9]
CA19.9 in U/mL, median [IQR]	88.7 [34.6–439] §	38.2 [24–169]	215.7 [58.0–580.5]	63.6 [39.1–142]	71.5 [31.0–332.2]
Number of EUS procedures per patient—*n* (%)					
One	122 (90.4)	11 (91.7)	58 (89.2)	12 (92.3)	41 (91.1)
Two	11 (8.1)	1 (8.3)	5 (7.7)	1 (7.7)	4 (8.9)
Three	2 (1.5)	0 (0)	2 (3.1)	0 (0)	0 (0)
Cross-sectional Imaging—*n* (%)					
CT	57 (42.2)	5 (41.7)	25 (38.5)	4 (30.8)	23 (51.1)
MRI	93 (68.9)	7 (58.3)	52 (80.0)	7 (53.9)	27 (60)
PET					
o FDG	38 (28.1)	3 (25.0)	20 (30.8)	4 (30.8)	11 (24.4)
o FDG + FAPI	8 (5.9)	0 (0)	6 (9.2)	1 (7.7)	1 (2.2)
None	10 (7.4)	2 (16.7)	3 (4.6)	1 (7.7)	4 (8.9)
Prior cholecystectomy—*n* (%)	23 (17.0)	2 (16.7)	14 (21.5)	4 (30.8)	3 (6.7)
**Based on pre-EUS cross-sectional imaging**					
Lymphadenopathy in report—*n* (%)					
Regional LN	58 (43.7)	4 (33.3)	38 (58.5)	6 (46.2)	11 (24.4)
Extraregional LN	37 (27.4)	6 (66.7)	21 (32.3)	5 (38.5)	5 (11.1)
Location—*n* (%)	NA		NA	NA	NA
Left		5 (41.7)			
Right		6 (50.0)			
Central		1 (8.3)			
**Based on surgery**					
Surgery—*n* (%)	84 (62.2)	8 (66.7)	33 (50.8)	6 (46.2)	37 (82.2)
Diagnostic laparoscopy	3 (2.2)	1 (8.3)	2 (3.1)	0 (0)	0 (0)
Explorative laparotomy	10 (7.4)	0 (0)	8 (12.3) ¶	1 (7.7) *	1 (2.2)
Resection	71 (52.6)	7 (58.3)	23 (35.4)	5 (38.5)	36 (80) **
pN stage in resection specimens—*n* (%) £					
N0	27 (38.0)	4 (57.1)	6 (26.1)	4 (80.0)	12 (33.3)
N1	22 (31.0)	2 (28.6)	6 (26.1)	1 (20.0)	14 (38.9)
N2	8 (11.3)	0 (0)	0 (0)	0 (0)	8 (22.2)
Nx	8 (11.3)	1 (14.3)	7 (30.4)	0 (0)	0 (0)
Benign disease—*n* (%)	6 (8.5)	0 (0)	4 (17.4)	0 (0)	2 (5.6)

* = palliative hepaticojejunostomy; ** = in one patient DLS and resection in separate procedure with neo-adjuvant chemotherapy before resection, and in another separate explorative laparotomy followed by resection; ¥ = missing in 4 patients (iCCA: 1, pCCA: 1, dCCA: 2); § = missing in 14 patients (iCCA: 3, pCCA: 9, dCCA: 2); ¶ = due to positive periportal (regional) LN in one patient, and due to positive paraaortic (extraregional) LN in another; £ = patients who underwent resection as denominator; NA = Not Applicable.

**Table 3:** Characteristics of the EUS procedures per procedure and outcomes per patient.

Variable (per EUS Procedure)	Total (*n* = 148)	iCCA (*n* = 13)	pCCA (*n* = 72)	mCCA (*n* = 14)	dCCA (*n* = 49)
Location of EUS—*n* (%)					
Treatment center	144 (97.3)	13 (100)	72 (100)	13 (92.9)	46 (93.9)
Referring hospital	4 (2.7)	0 (0)	0 (0)	1 (7.1)	3 (6.1)
Drainage procedure prior to EUS—*n* (%)					
ERCP plastic stent	35 (23.6)	0 (0)	19 (26.4)	3 (21.4)	13 (26.5)
ERCP metal stent	2 (1.4)	0 (0)	1 (1.4)	0 (0)	1 (2.0)
PTBD	2 (1.4)	1 (7.7)	1 (1.4)	0 (0)	0 (0)
Cholangitis within 30 days of EUS—*n* (%)	3 (2.0)	0 (0)	2 (2.8)	0 (0)	1 (2.0)
Post-ERCP pancreatitis within 30 days of EUS—*n* (%)	2 (1.4)	0 (0) a	1 (1.4)	0 (0)	1 (2.0)
During EUS other TA—*n* (%)					
FNA mass	17 (11.5)	1 (7.7)	5 (6.9)	2 (14.3)	9 (18.4)
FNB mass	21 (14.2)	2 (15.4)	9 (12.5) b	1 (7.1)	9 (18.4)
Liver lesion	1 (0.7)	0 (0)	1 (1.4) c	0 (0)	0 (0)
≥1 LN described at EUS—*n* (%)					
Regional	66 (44.6)	3 (23.1)	38 (52.8)	7 (50.0)	18 (36.7)
Extraregional	11 (7.4)	3 (23.1)	4 (5.6)	1 (7.1)	3 (6.1)
Both	17 (11.5)	5 (38.5)	10 (13.4)	1 (7.1)	1 (2.0)
Complication—*n* (%)	0 (0)	0 (0)	0 (0)	0 (0)	0 (0)
**Variable (per patient)**	**Total** **(*n* = 135)**	**iCCA** **(*n* = 12)**	**pCCA** **(*n* = 65)**	**mCCA** **(*n* = 13)**	**dCCA** **(*n* = 45)**
EUS-TA of LN—*n* (%)					
Regional	44 (32.6)	3 (25.0)	29 (44.6)	3 (23.1)	9 (20)
Extraregional	9 (6.7)	2 (16.7)	6 (9.2)	1 (7.7)	0 (0)
Both	3 (2.2)	2 (16.7)	1 (1.5)	0 (0)	0 (0)
Positive LN with EUS-TA—*n* (%)					
Regional	7 (5.2)	1 (8.3)	3 (4.6)	1 (7.7)	2 (4.4)
Extraregional	3 (2.2)	1 (8.3)	1 (1.5)	1 (7.7)	0 (0)
Both	1 (0.7)	1 (8.3)	0 (0)	0 (0)	0 (0)
EUS precluding surgical work-up—*n* (%)					
Regional LN positive	1 (0.7)	1 (8.3)	0 (0)	0 (0)	0 (0)
Extraregional LN positive	2 (1.5)	1 (8.3)	0 (0)	1 (7.7)	0 (0)
Regional and extraregional LN positive	2 (1.5)	1 (8.3)	1 (1.5)	0 (0)	0 (0)
Superior mesenteric artery involvement	1 (0.7)	0 (0)	0 (0)	1 (7.7)	0 (0)

a = one patient with known chronic pancreatitis; b = one patient had two times FNB of mass; c = FNA of benign liver lesion.

**Table 4:** Characteristics of the described LN.

Described LN	#	EUS-TA			Pathology Results		
		FNA	FNB	Not Performed	Malignant	Benign	Atypical Cells
Regional	106	24	24	58 *	8	39	1
Extraregional	33	9	6	18 **	4	11	0
Total	139	33	30	76	12	50	1

* = not safely possible (*n* = 3); other LN biopsied during EUS (*n* = 10); not suspicious (*n* = 40); suspicious but no EUS-TA for unclear reason (*n* = 5). ** = not safely possible (*n* = 1); other LN biopsied during EUS (*n* = 6); not suspicious (*n* = 9); suspicious but no EUS-TA for unclear reason (*n* = 2).

## Data Availability

The data that support the findings of this study are available upon reasonable request from the corresponding authors.

## Supplementary Materials

The following supporting information can be downloaded at https://www.mdpi.com/article/10.3390/jcm14217545/s1. Supplementary Table S1. Information regarding ‘missed’ LN, Supplementary Figure S1. Flowchart for iCCA, Supplementary Figure S2. Flowchart for pCCA, Supplementary Figure S3. Flowchart for mCCA, Supplementary Figure S4. Flowchart for dCCA. Supplemental Text S1. FAPI findings.

## Abbreviations

The following abbreviations are used in this manuscript:

LN	Lymph node
CCA	Cholangiocarcinoma
EUS	Endoscopic ultrasound
EUS-TA	EUS-guided tissue acquisition
iCCA	Intrahepatic Cholangiocarcinoma
pCCA	Perihilar Cholangiocarcinoma
mCCA	Middle bile duct Cholangiocarcinoma
dCCA	Distal Cholangiocarcinoma
AJCC	American Joint Committee on Cancer
CT	Computed tomography
MRI	Magnetic resonance imaging
ESGE	European Society of Gastrointestinal Endoscopy
ERCP	Endoscopic Retrograde Cholangio-pancreatography
EASL	European Association for the Study of the Liver
FDG	Fluorodeoxyglucose
PET	Positron emission tomography
FAPI	Fibroblast-activation-protein inhibitors
SUV	Standardized uptake value
FNA	Fine-needle aspiration
FNB	Fine-needle biopsy
ROSE	Rapid on-site evaluation
ASA	American Society of Anesthesiologists’ Physical Status Classification System
PSC	Primary Sclerosing Cholangitis
IQR	Interquartile range
PTBD	Percutaneous Trans-hepatic Biliary Drainage

## References

[1] Blechacz B. (2017). Cholangiocarcinoma: Current Knowledge and New Developments. Gut Liver.

[2] Zhang X.F., Xue F., Dong D.H., Weiss M., Popescu I., Marques H.P., Aldrighetti L., Maithel S.K., Pulitano C., Bauer T.W. (2021). Number and Station of Lymph Node Metastasis After Curative-intent Resection of Intrahepatic Cholangiocarcinoma Impact Prognosis. Ann. Surg..

[3] van Keulen A.-M., Buettner S., Erdmann J.I., Pratschke J., Ratti F., Jarnagin W.R., Schnitzbauer A.A., Lang H., Ruzzenente A., Nadalin S. (2023). Multivariable prediction model for both 90-day mortality and long-term survival for individual patients with perihilar cholangiocarcinoma: Does the predicted survival justify the surgical risk?. Br. J. Surg..

[4] Strijker M., Belkouz A., van der Geest L.G., van Gulik T.M., van Hooft J.E., de Meijer V.E., Haj Mohammad N., de Reuver P.R., Verheij J., de Vos-Geelen J. (2019). Treatment and survival of resected and unresected distal cholangiocarcinoma: A nationwide study. Acta Oncol..

[5] Yee E.J., Ziogas I.A., Moris D.P., Torphy R.J., Mungo B., Gleisner A.L., Del Chiaro M., Schulick R.D. (2024). Cholangiocarcinoma of the Middle Bile Duct: A Narrative Review. Ann. Surg. Oncol..

[6] Schreuder A.M., Engelsman A.F., van Roessel S., Verheij J., Besselink M.G., van Gulik T.M., Busch R.O. (2019). Treatment of mid-bile duct carcinoma: Local resection or pancreatoduodenectomy?. Eur. J. Surg. Oncol..

[7] Amin M.B., Greene F.L., Edge S.B., Compton C.C., Gershenwald J.E., Brookland R.K., Meyer L., Gress D.M., Byrd D.R., Winchester D.P. (2017). The Eighth Edition AJCC Cancer Staging Manual: Continuing to build a bridge from a population-based to a more “personalized” approach to cancer staging. CA Cancer J. Clin..

[8] Nooijen L.E., Banales J.M., de Boer M.T., Braconi C., Folseraas T., Forner A., Holowko W., Hoogwater F.J.H., Klümpen H.-J., Groot Koerkamp B. (2022). Impact of Positive Lymph Nodes and Resection Margin Status on the Overall Survival of Patients with Resected Perihilar Cholangiocarcinoma: The ENSCCA Registry. Cancers.

[9] Lee H.G., Lee S.H., Do Yoo D., Paik K.Y., Heo J.S., Choi S.H., Choi D.W. (2009). Carcinoma of the middle bile duct: Is bile duct segmental resection appropriate?. World J. Gastroenterol..

[10] Lyu S., Li L., Zhao X., Ren Z., Cao D., He Q. (2020). Prognostic impact of lymph node parameters in distal cholangiocarcinoma after pancreaticoduodenectomy. World J. Surg. Oncol..

[11] Nishioka E., Tsurusaki M., Kozuki R., Im S.W., Kono A., Kitajima K., Murakami T., Ishii K. (2022). Comparison of Conventional Imaging and 18F-Fluorodeoxyglucose Positron Emission Tomography/Computed Tomography in the Diagnostic Accuracy of Staging in Patients with Intrahepatic Cholangiocarcinoma. Diagnostics.

[12] Ruys A.T., Van Beem B.E., Engelbrecht M.R.W., Bipat S., Stoker J., van Gulik T.M. (2014). Radiological staging in patients with hilar cholangiocarcinoma: A systematic review and meta-analysis. Br. J. Radiol..

[13] Hänninen E.L., Pech M., Jonas S., Ricke J., Thelen A., Langrehr J., Hintze R., Röttgen R., Denecke T., Winter L. (2005). Magnetic resonance imaging including magnetic resonance cholangiopancreatography for tumor localization and therapy planning in malignant hilar obstructions. Acta Radiol..

[14] Yoo J., Lee J.M., Kang H.J., Bae J.S., Jeon S.K., Yoon J.H. (2023). Comparison Between Contrast-Enhanced Computed Tomography and Contrast-Enhanced Magnetic Resonance Imaging with Magnetic Resonance Cholangiopancreatography for Resectability Assessment in Extrahepatic Cholangiocarcinoma. Korean J. Radiol..

[15] Gleeson F.C., Rajan E., Levy M.J., Clain J.E., Topazian M.D., Harewood G.C., Papachristou G.I., Takahashi N., Rosen C.B., Gores G.J. (2008). EUS-guided FNA of regional lymph nodes in patients with unresectable hilar cholangiocarcinoma. Gastrointest. Endosc..

[16] Malikowski T., Levy M.J., Gleeson F.C., Storm A.C., Vargas E.J., Topazian M.D., Abu Dayyeh B.K., Iyer P.G., Rajan E., Gores G.J. (2020). Endoscopic Ultrasound/Fine Needle Aspiration Is Effective for Lymph Node Staging in Patients with Cholangiocarcinoma. Hepatology.

[17] de Jong D.M., van de Vondervoort S., Dwarkasing R.S., Thomeer M.G.J., Doukas M., Voermans R.P., Verdonk R.C., Polak W.G., de Jonge J., Bruno M.J. (2024). Endoscopic ultrasound with tissue acquisition of lymph nodes in patients with potentially resectable intrahepatic cholangiocarcinoma. Endosc. Int. Open.

[18] de Jong D.M., van de Vondervoort S., Dwarkasing R.S., Doukas M., Voermans R.P., Verdonk R.C., Polak W.G., de Jonge J., Groot Koerkamp B., Bruno M.J. (2023). Endoscopic ultrasound in patients with resectable perihilar cholangiocarcinoma: Impact on clinical decision-making. Endosc. Int. Open.

[19] de Jong D.M., den Hoed C.M., Willemssen F.E.J.A., Thomeer M.G.J., Bruno M.J., Groot Koerkamp B., de Jonge J., Alwayn I.P.J., van Hooft J.E., Hoogwater F. (2024). Impact of EUS in liver transplantation workup for patients with unresectable perihilar cholangiocarcinoma. Gastrointest. Endosc..

[20] Mohamadnejad M., DeWitt J.M., Sherman S., LeBlanc J.K., Pitt H.A., House M.G., Jones K.J., Fogel E.L., McHenry L., Watkins J.L. (2011). Role of EUS for preoperative evaluation of cholangiocarcinoma: A large single-center experience. Gastrointest. Endosc..

[21] Sato K., Shigekawa M., Yamamoto S., Matsumae T., Sato Y., Yoshioka T., Kodama T., Hikita H., Tatsumi T., Takehara T. (2025). Utility and clinical significance of endoscopic ultrasound-guided tissue acquisition for diagnosing lymphadenopathies in biliary tract cancer. Sci. Rep..

[22] Fritscher-Ravens A., Broering D.C., Sriram P.V., Topalidis T., Jaeckle S., Thonke F., Soehendra N. (2000). EUS-guided fine-needle aspiration cytodiagnosis of hilar cholangiocarcinoma: A case series. Gastrointest. Endosc..

[23] Rai P., Kumar V., Rao R.N. (2017). Malignant mediastinal lymphadenopathy detected by endoscopic ultrasound and guided fine needle aspiration in patients with resectable pancreaticobiliary cancer. Indian J. Gastroenterol..

[24] Facciorusso A., Crinò S.F., Gkolfakis P., Spadaccini M., Arvanitakis M., Beyna T., Bronswijk M., Dhar J., Ellrichmann M., Gincul R. (2024). Diagnostic work-up of bile duct strictures: European Society of Gastrointestinal Endoscopy (ESGE) Guideline. Endoscopy.

[25] Marzioni M., Maroni L., Aabakken L., Carpino G., Groot Koerkamp B., Heimbach J., Khan S., Lamarca A., Saborowski A., Vilgrain V. (2025). EASL Clinical Practice Guidelines on the management of extrahepatic cholangiocarcinoma. J. Hepatol..

[26] de Jong D.M., Lammers W.J., van Driel L.M.J.W. (2025). Time to standardize preoperative EUS for lymph node staging in resectable extrahepatic cholangiocarcinoma. J. Hepatol..

[27] Nass K.J., Zwager L.W., van der Vlugt M., Dekker E., Bossuyt P.M.M., Ravindran S., Thomas-Gibson S., Fockens P. (2022). Novel classification for adverse events in GI endoscopy: The AGREE classification. Gastrointest. Endosc..

[28] de Jong D.M., Roosterman D., Bruno M.J., van Driel L.M.J.W., Lammers W.J. (2025). Interobserver variability in lymph node evaluation with endoscopic ultrasonography in cholangiocarcinoma. Endosc. Int. Open.

[29] Liang L., Li C., Jia H.D., Diao Y.K., Xing H., Pawlik T.M., Lau W.Y., Shen F., Huang D.S., Zhang C.W. (2021). Prognostic factors of resectable perihilar cholangiocarcinoma: A systematic review and meta-analysis of high-quality studies. Ther. Adv. Gastrointest. Endosc..

[30] Parente A., Kamarajah S.K., Baia M., Tirotta F., Manzia T.M., Hilal M.A., Pawlik T.M., White S.A., Dahdaleh F.S. (2023). Neoadjuvant Chemotherapy for Intrahepatic, Perihilar, and Distal Cholangiocarcinoma: A National Population-Based Comparative Cohort Study. J. Gastrointest. Surg..

[31] de Jong D.M., van de Vondervoort S., Dwarkasing R.S., Doukas M., Voermans R.P., Verdonk R.C., Polak W.G., de Jonge J., Groot Koerkamp B., Bruno M.J. (2025). Endoscopic ultrasound with tissue acquisition of lymph nodes in patients with resectable distal cholangiocarcinoma. Scand. J. Gastroenterol..

[32] Terasaki F., Sugiura T., Okamura Y., Ashida R., Ohgi K., Yamada M., Ohtsuka S., Uesaka K. (2024). Benefit of lymph node dissection for perihilar and distal cholangiocarcinoma according to lymph node stations. J. Hepato-Biliary-Pancreat. Sci..

[33] de Jong D.M., Chehin K., Meijering T.L.N., Segbers M., van Driel L.M.J.W., Bruno M.J., Groot Koerkamp B., IJzermans J.N.M., Verburg F.A., de Lussanet de la Sabloniere Q.G. (2024). Hybrid FDG-PET/MRI for Diagnosis and Clinical Management of Patients with Suspected Perihilar Cholangiocarcinoma: A Feasibility Pilot Study. Nucl. Med. Mol. Imaging.

[34] Veldhuijzen van Zanten S.E.M., Pieterman K.J., Wijnhoven B.P.L., Pruis I.J., Groot Koerkamp B., van Driel L., Verburg F.A., Thomeer M.G.J. (2022). FAPI PET versus FDG PET, CT or MRI for Staging Pancreatic-, Gastric- and Cholangiocarcinoma: Systematic Review and Head-to-Head Comparisons of Diagnostic Performances. Diagnostics.

