# Supplementary material for: Endoscopic Ultrasound for Nodal Staging in Patients with Resectable Cholangiocarcinoma
Source: J Clin Med. 2025 Oct 24;14(21):7545. doi: 10.3390/jcm14217545 (PMC12609811; doi:10.3390/jcm14217545)
Supplement: Supplementary file 1 [file jcm-14-07545-s001.zip › jcm-3910172-supplementary.pdf]

Supplementary files

#	CCA subtype	LN at EUS identified	EUS-TA of which LN	Location of positive LN at surgery (number of LN)	Missed on EUS?
1	pCCA	Periportal, hepato-gastric & hepatic hilum	Periportal	Periportal/HDL (2)	Biopsied
2	dCCA	Pericholedochal, peripancreatic, periportal, hepatic hilum	Hepatic hilum, periportal	Pancreatic (5)	Seen
3	dCCA	Periportal	Periportal	Peripancreatic (3)	Possible
4	pCCA	Portocaval	Portocaval	Hilar (1)	Possible
5	pCCA	Periportal, portal vein	Periportal	Retro-pancreatic	Possible
6	pCCA	Periportal, aortocaval, mediastinal	Mediastinal	HDL (2), common hepatic (1)	Possible
7	iCCA	Paraaortic	Paraaortic	HDL/para-caval (1)	Not seen
8	dCCA	Pericholedochal	Pericholedochal	Gastric (1)	Not seen
9	dCCA	Peripancreatic	x	Peripancreatic (9)	Seen
10	CBD	Hepatic hilum	x	Bile duct (3)	Seen
11	iCCA	Periportal	x	Periportal (1), CBD (1)	Seen
12	dCCA	Periportal, aortocaval	x	Peripancreatic (2)	Possible
13	pCCA	Periportal	x	HDL (1)	Possible
14	dCCA	Periportal	x	Peripancreatic (1)	Possible
15	dCCA	Periportal	x	CBD (1)	Possible
16	dCCA	Periportal	x	'Regional' (3)	Possible
17	dCCA	Pericholedochal	x	Peripancreatic (1)	Possible
18	pCCA	Periportal, porta-caval & aorto-caval	Periportal	Periportal (2)	Biopsied

Supplementary Table S1. Information regarding 'missed' LN

In the remaining twelve patients at EUS no LN were described.

CCA = cholangiocarcinoma, LN = lymph node, EUS = endoscopic ultrasound, TA = tissue acquisition, iCCA = intrahepatic cholangiocarcinoma, pCCA = perihilar cholangiocarcinoma, mCCA = middle bile duct cholangiocarcinoma, dCCA = distal cholangiocarcinoma, HDL = hepatoduodenal ligament, CBD = common bile duct

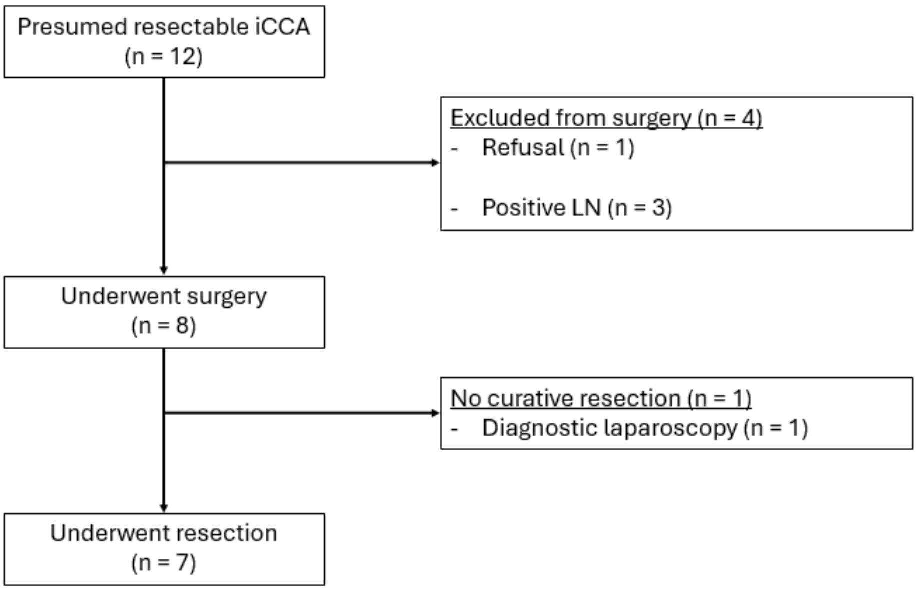

<u>Supplementary Figure S1.</u> Flowchart for iCCA

No patients with positive LN continued to surgery

iCCA = intrahepatic cholangiocarcinoma, LN = lymph node

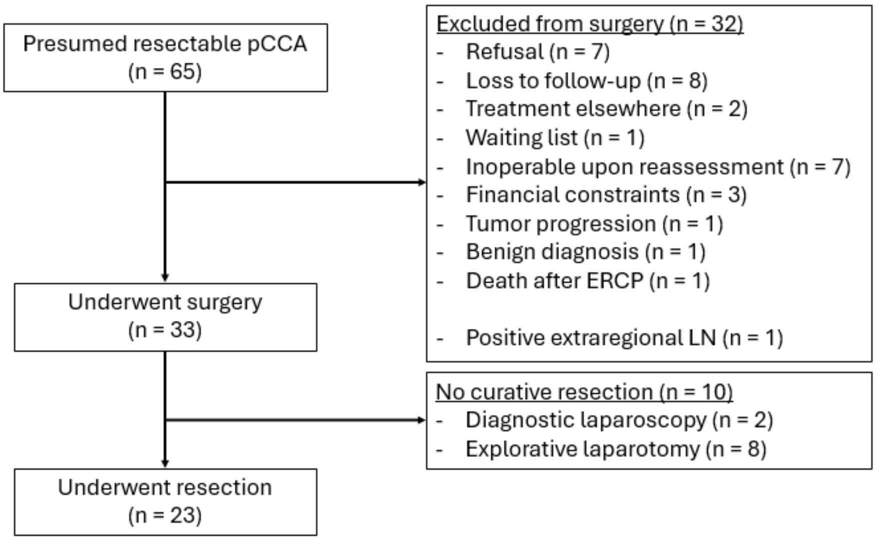

<u>Supplementary Figure S2.</u> Flowchart for pCCA

Three patients with positive regional LN were worked up for surgery: one had tumor progression on CT, one underwent diagnostic laparoscopy without resection due to cirrhotic liver disease, and one patient underwent hepatectomy showing pT2bN1 pCCA.

pCCA: perihilar cholangiocarcinoma, FU = follow-up, ERCP = Endoscopic retrograde cholangiopancreatography, DLS = Diagnostic laparoscopy

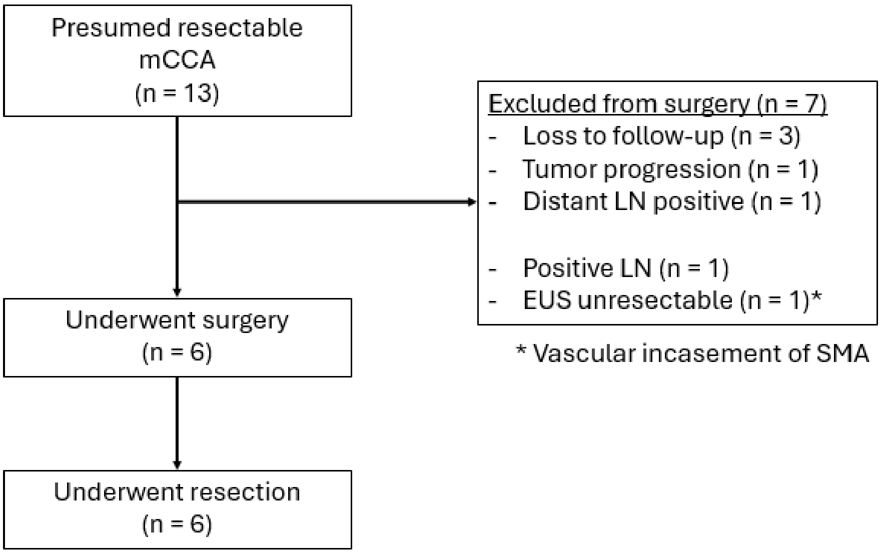

Supplementary Figure S3. Flowchart for mCCA

One patient with positive regional LN was lost to follow-up, probably to receive surgery elsewhere (defined as loss to FU).

mCCA = middle bile duct cholangiocarcinoma, LN = lymph node, EUS = Endoscopic ultrasound, SMA = superior mesenteric artery

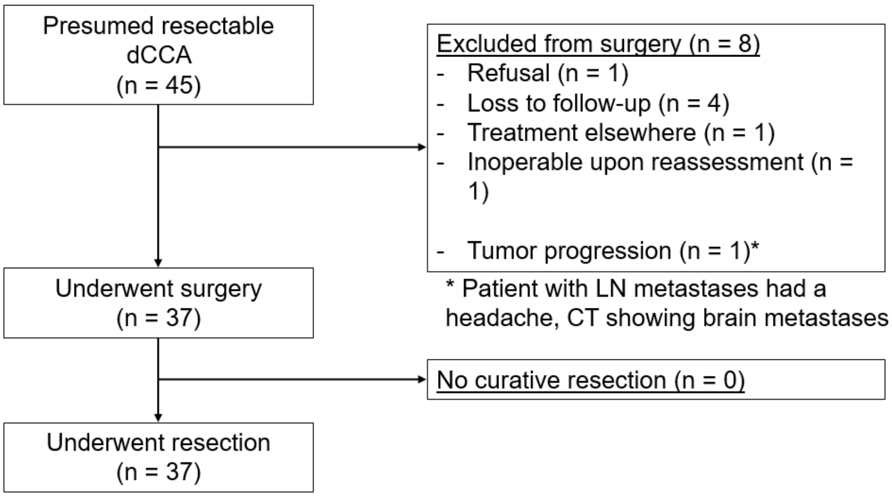

Supplementary Figure S4. Flowchart for dCCA

One patient with positive regional LN underwent resection showing a dCCA pT3N2

dCCA = distal cholangiocarcinoma, LN = lymph node, CT = computed tomography

Supplemental text S1. FAPI findings

In 18 patients FAPI and FDG PET scan (13 pCCA, 1 iCCA, 1 mCCA, 3 dCCA), of which 10 before EUS, 6 after EUS and 2 between first and 2nd EUS procedures. In 10 patients these scans showed no LN.

In eight patients LN were identified, and all these LN were biopsied during EUS.

- Only malignant LN with EUS-TA was strongly PET avid with FDG and FAPI.
- Four patients had non FDG and FAPI avid LN, all were benign
- Three patients had mild-to-moderate FDG avid LN, but showed no FAPI uptake, all were benign.

Only 5 patients underwent resection (+1 patient with DLS showing peritoneal metastasis):

- One patient with FDG avid but no FAPI uptake in periportal LN had N0 disease
- Four patients with no LN on FDG/FAPI
    - Two had N0, one had Nx
    - One patient had N1 (3/3 malignant peripancreatic LN)
